# Supplementary material for: Single nucleus sequencing reveals spermatid chromosome fragmentation as a possible cause of maize haploid induction
Source: Nat Commun. 2017 Oct 23;8:991. doi: 10.1038/s41467-017-00969-8 (PMC5653662; doi:10.1038/s41467-017-00969-8)
Supplement: Supplementary file 1 — Supplementary information [file 41467_2017_969_MOESM1_ESM.pdf]

Supplementary Figure 1

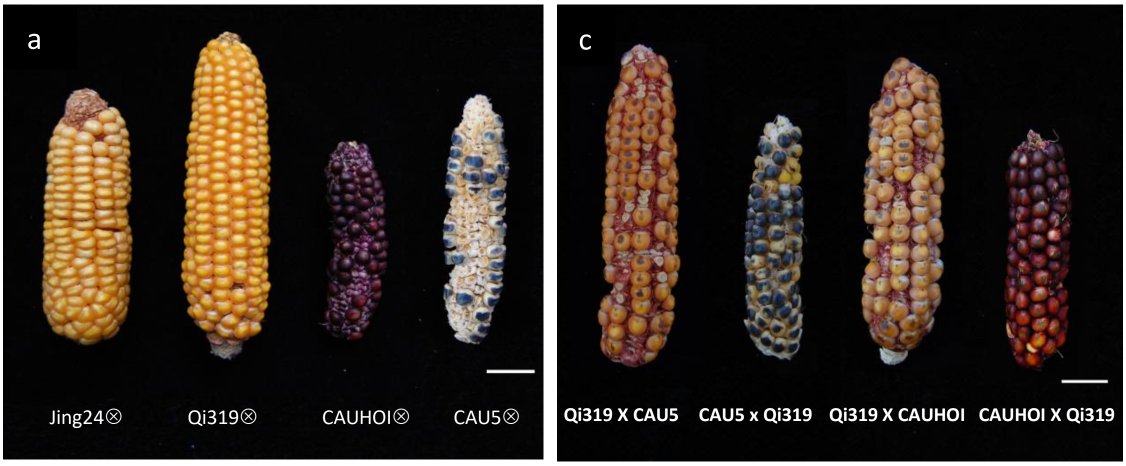

b

♀ / ♂	Qi319	Jing24	CAU5	CAUHOI
**Qi319**	**3.33%**	**X**	**0%**	**2.66%**
**Jing24**	**X**	**2.45%**	**0%**	**1.76%**
**CAU5**	**9.26%**	**9.61%**	**56.55%**	**X**
**CAUHOI**	**2.98%**	**2.01%**	**X**	**10.76%**

Supplementary Figure 1. Feature of selfing ears and crossing ears in four lines. (a) self- and cross-pollinated ears from four different genotypes/crosses. (b) The percentage of aborted kernels in selfing ears and crossing ears. Scale bar = 2.5cm. The Numbers of the detected kernels from selfing ears are 1174 (for Jing24⊗), 1786 (for Qi319⊗), 1335 (for CAUHOI⊗), 688 (for CAU5⊗) . (c) Crossing ears of four lines. The Numbers of the detected kernels from crossing ears are 1360 (for Qi319X CAU5), 1235 (for CAU5 X Qi319), 1140 (for Qi319 X CAUHOI), 1201 (for CAUHOI X Qi319) .

Supplementary Figure 2

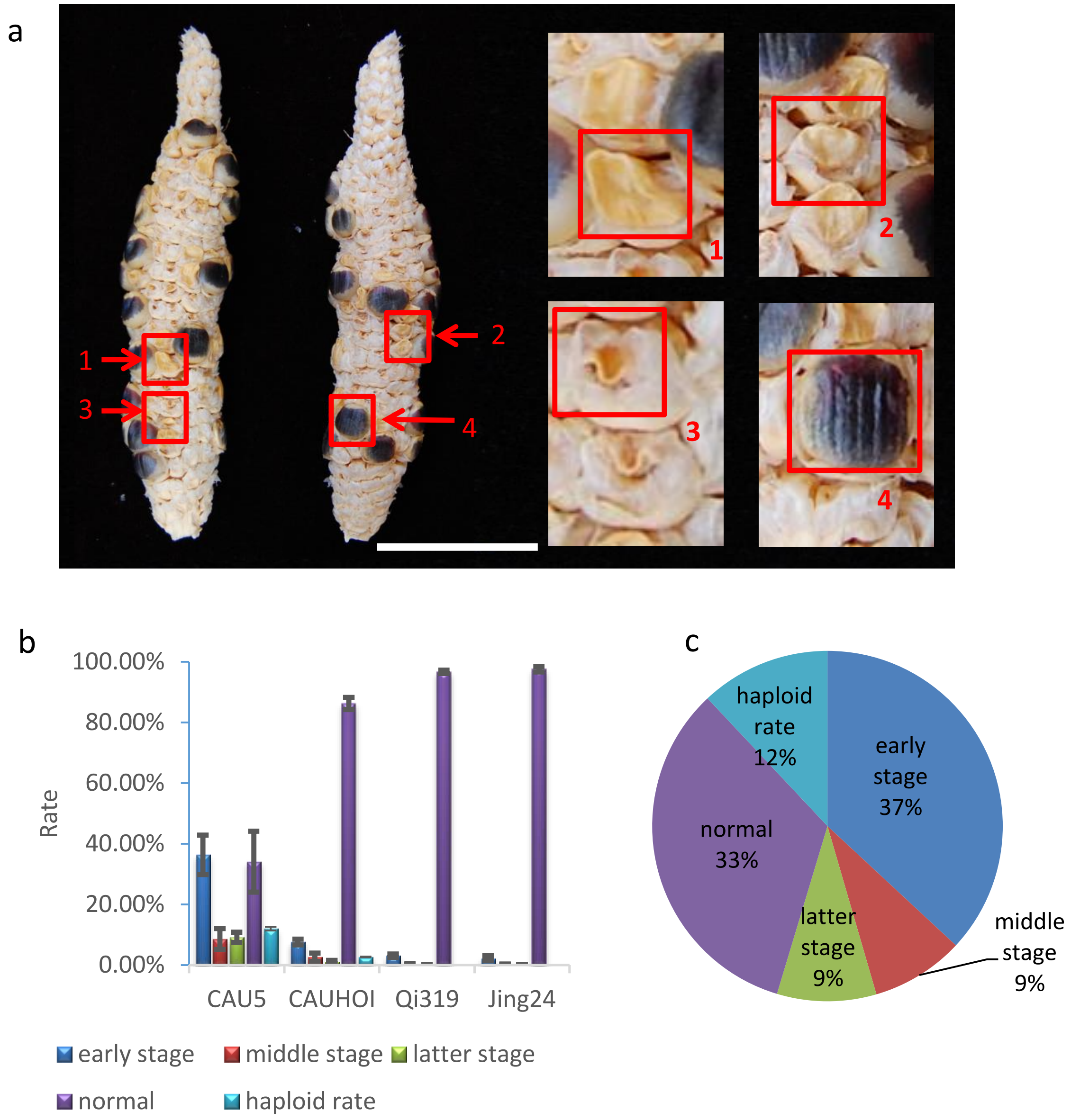

Supplementary Figure 2. Grade specification of abortion kernels in selfing ears of four lines. (a) A feature of CAU5 selfing ears (bar = 2.5 cm). Symbols 1 to 4 represent kernels with abortion in the latter, median, early developmental stages, and a normal kernel, respectively. (b) The kernel composition of selfing ears in the four lines. Error bars represent standard deviations (SD). (c) A feature of the kernel composition of CAU5 selfing ears. The Numbers of the detected kernels are 688 (for CAU5), 788 (for CAUHOI), 862 (for Qi319), 814 (for Jing24) .

Supplementary Figure 3

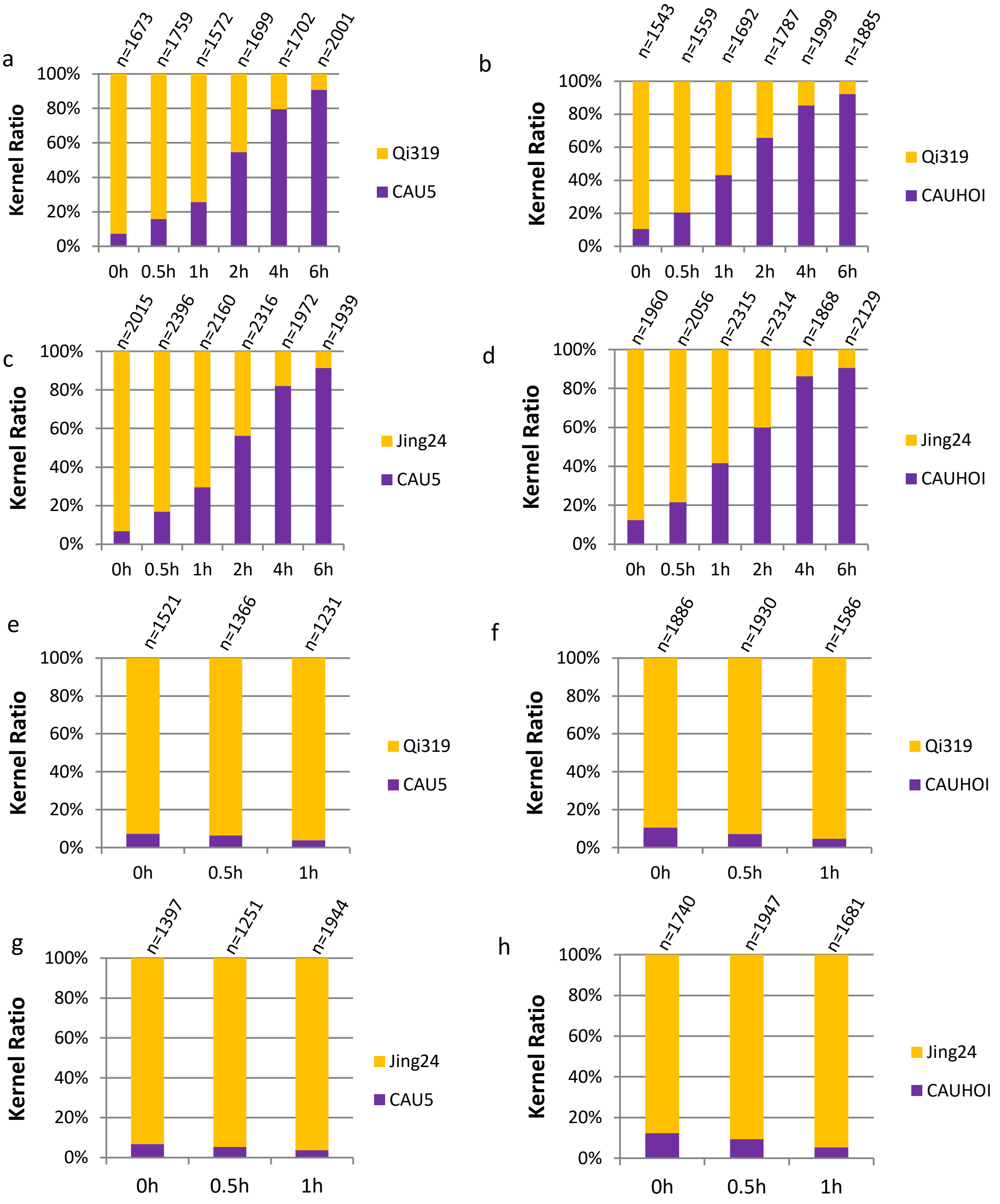

Supplementary Figure 3. Pollen competitive ability. (a-d) The ears pollinated first with pollen from the inducer lines were pollinated again with pollen from inbred lines during different pollination intervals (0-6h). (e-h) The ears pollinated first with pollen from the inbred lines were pollinated again with pollen from inducer lines in 0-1h pollination intervals. Taking CAU5 as an example, when female parent were crossed with simultaneous pollination with a 1:1 mixture of inducer and non-inducer pollen, up to 92.7% of the resulting kernels had colorless endosperm, even though prolonged the pollination interval time to 6h, the colorless endosperm was still nearly 10%. Taking an overall view of the six intervals, focusing on the CAU5 harvested ears of the 2h pollination interval, the ratio of purple kernels/non-purple kernels was nearly 1:1, which demonstrated that with the CAU5 pollinated 2h earlier, the PCA is similar to that of the inbred lines.

Supplementary Figure 4

a

c

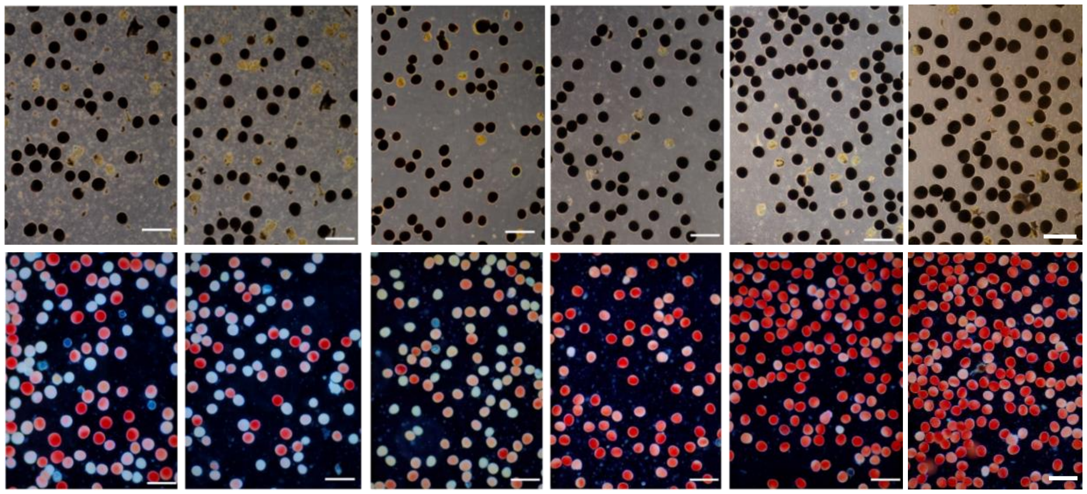

CAU5 B73-inducer CAUHOI Qi319 Jing24 B73

b

Fertility	Wild Type	Spherical abortion	Typical abortion
CAU5	79.1±1.6%	1.9±0.6%	19.0±1.2%
B73-inducer	81.3±1.4%	2.0±0.4%	16.7±1.1%
CAUHOI	83.6±0.6%	2.9±0.7%	13.5±0.2%
Qi319	90.9±1.8%	0.1±0.1%	9.0±1.9%
Jing24	91.3±1.2%	0.1±0.1%	8.6±1.2%
B73	92.0±1.7%	0.3±0.1%	7.7±1.8%
ANOVA P	1.95406E-07	5.78756E-06	6.74403E-07

d

Viability	Abortion	None	Low	Medium	High
CAU5	18.2±0.7%	22.5±2.8%	30.1±1.8%	23.2±2.3%	6.0±0.6%
B73-inducer	17.0±0.2%	25.8±0.2%	28.8±1.3%	21.8±1.2%	6.7±0.4%
CAUHOI	13.2±3.2%	21.0±2.5%	27.8±1.1%	35.3±2.2%	2.9±0.3%
Qi319	9.0±0.8%	2.2±1.0%	5.5±1.3%	39.9±0.9%	43.4±1.0%
Jing24	8.4±0.2%	2.4±0.3%	4.6±1.1%	42.2±3.0%	42.5±2.3%
B73	8.0±1.8%	2.2±0.7%	6.0±0.4%	43.4±2.8%	40.4±1.7%
ANOVA P	5.86E-06	1.98E-10	1.88E-12	6.2E-08	1.62E-14

Supplementary Figure 4. Patterns of pollen fertility and viability. (a) Pollen fertility was detected via the KI stained pollen color. (b) Pollen viability could be detected by TTC staining. (Bar = 1mm) The distinct difference could be observed between the six samples. See Fig. 1 for detailed measurements. The percentages of fertility (c) and viability (d) levels has been shown to be diffenrent between lines for each level, confirmed by the significant ANOVA P value. Three biological repeats (individuals) was required for each line. Sample sizes are in Figure 1 of the main text.

Supplementary Figure 5: 5 pp.

Supplementary Figure 5. Aneuploidy of single sperm nuclei for B73, B73-inducer, Chang7-2 and CHOI3. (a) Sperm and trophic nuclei could be released, when one viable pollen was put in free water. The shape of trophic nucleus would be changed when through pollen hole. We isolated single sperm nuclei to sequence (Bar = 100 μm). The sequencing CNR plots of pollens in (b) B73, (c) B73-inducer, (d) Chang7-2 and (e) CHOI3, are shown that a whole genome elimination. The black lines mean the putative ploidy.

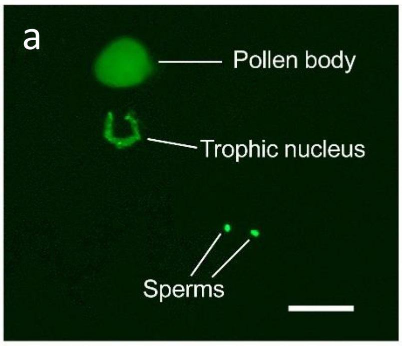

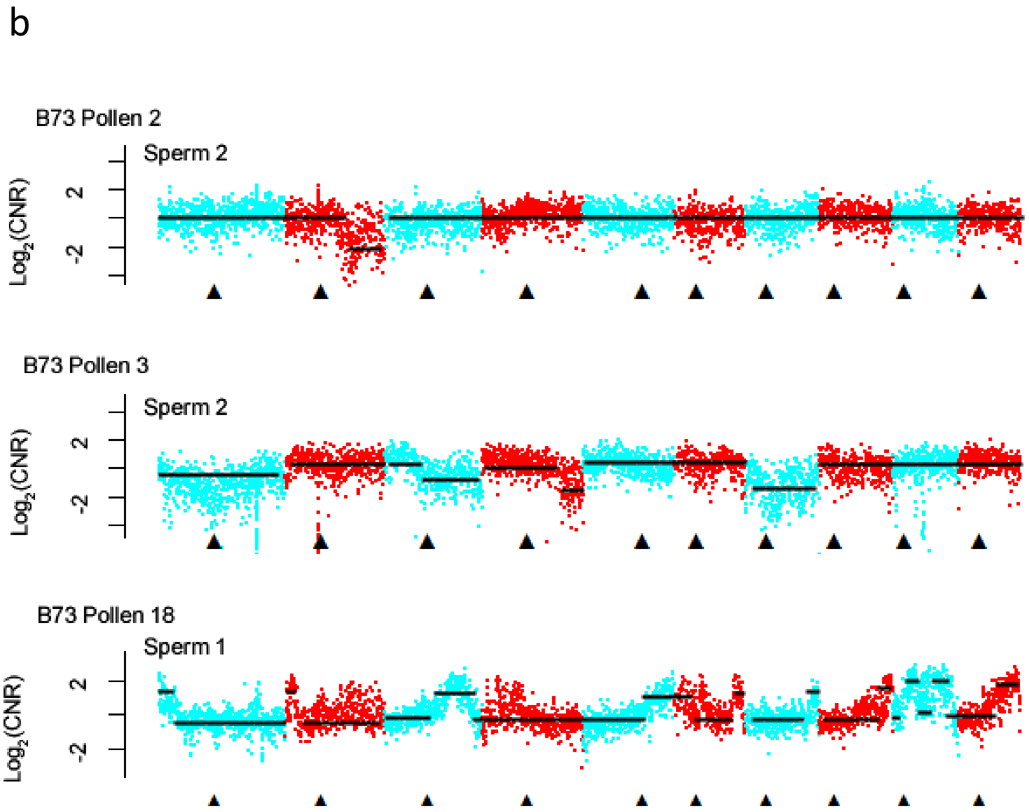

c

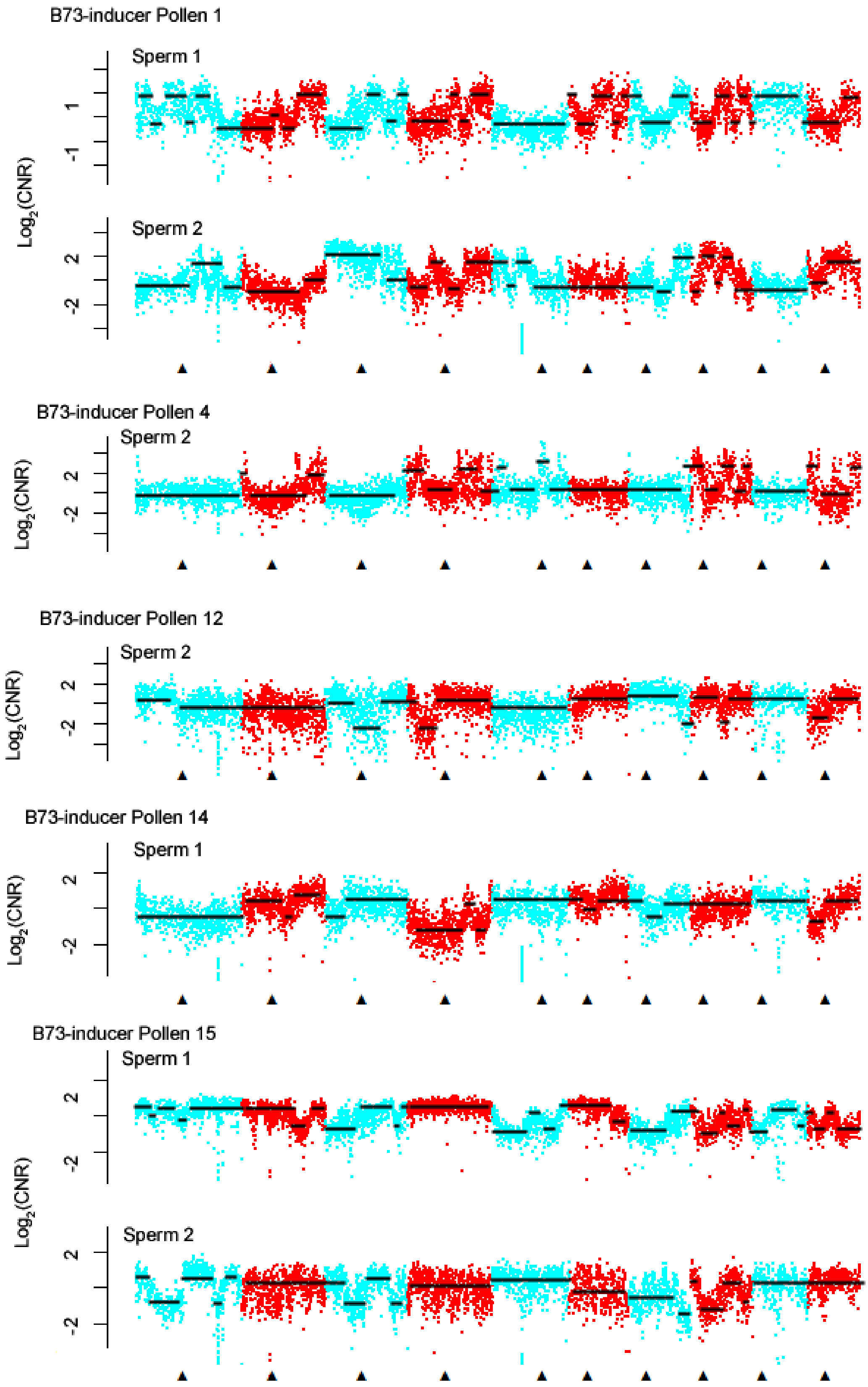

B73-inducer Pollen 1
Sperm 1
Sperm 2
Log2(CNR)
1
-1
2
-2
B73-inducer Pollen 4
Sperm 2
Log2(CNR)
2
-2
B73-inducer Pollen 12
Sperm 2
Log2(CNR)
2
-2
B73-inducer Pollen 14
Sperm 1
Log2(CNR)
2
-2
B73-inducer Pollen 15
Sperm 1
Sperm 2
Log2(CNR)
2
-2

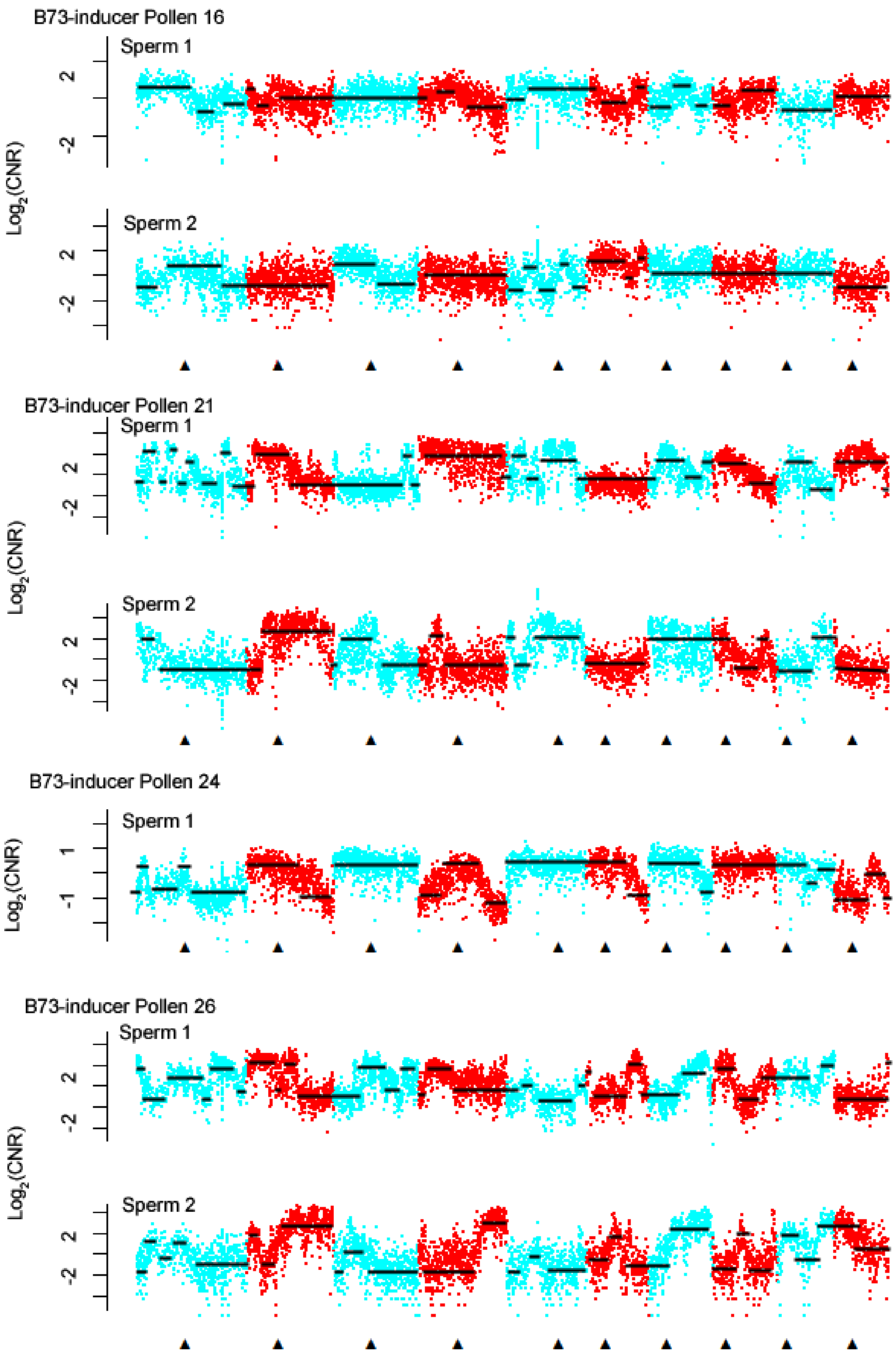

B73-inducer Pollen 16
Sperm 1
Sperm 2
Log2(CNR)
2
-2
B73-inducer Pollen 21
Sperm 1
Sperm 2
Log2(CNR)
B73-inducer Pollen 24
Sperm 1
1
-1
B73-inducer Pollen 26
Sperm 1
Sperm 2

d

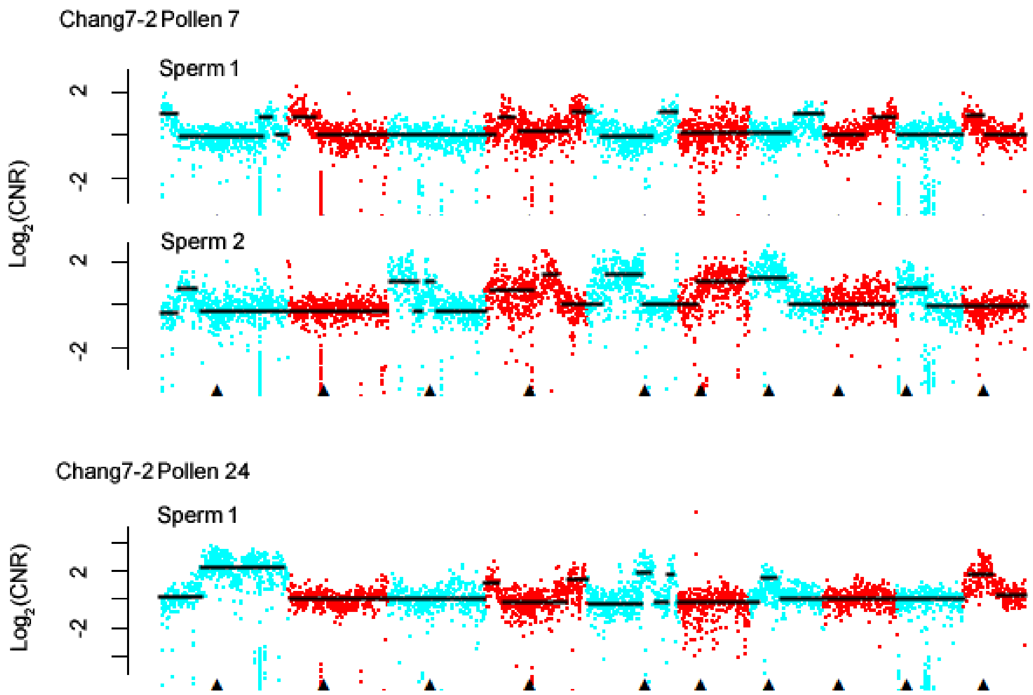
Chang7-2 Pollen 7
Sperm 1
Sperm 2
Log2(CNR)
2
-2
Chang7-2 Pollen 24
Sperm 1
Log2(CNR)
2
-2

e

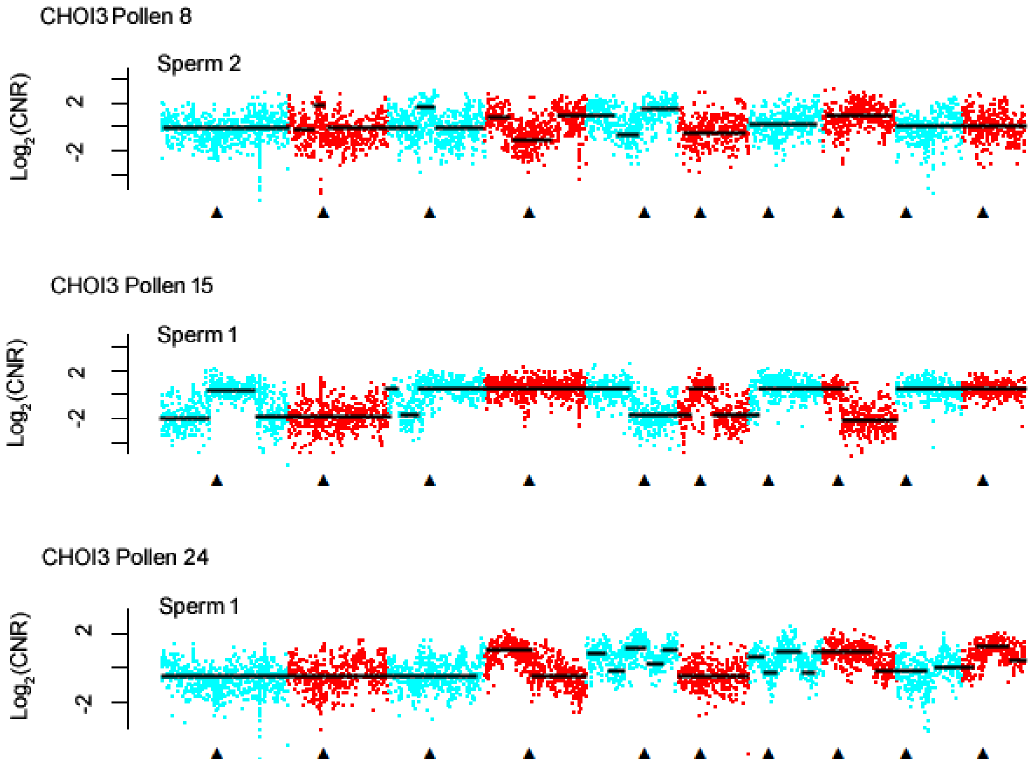
CHOI3 Pollen 8
Sperm 2
Log2(CNR)
2
-2
CHOI3 Pollen 15
Sperm 1
Log2(CNR)
2
-2
CHOI3 Pollen 24
Sperm 1
Log2(CNR)
2
-2

CHOI3 Pollen 30

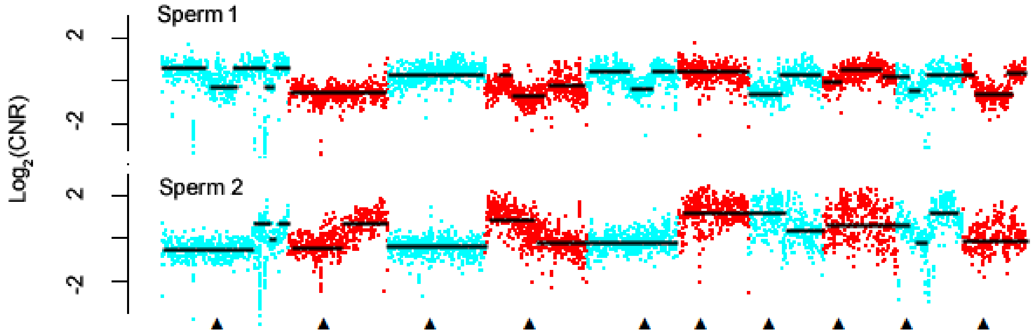

CHOI3 Pollen 34

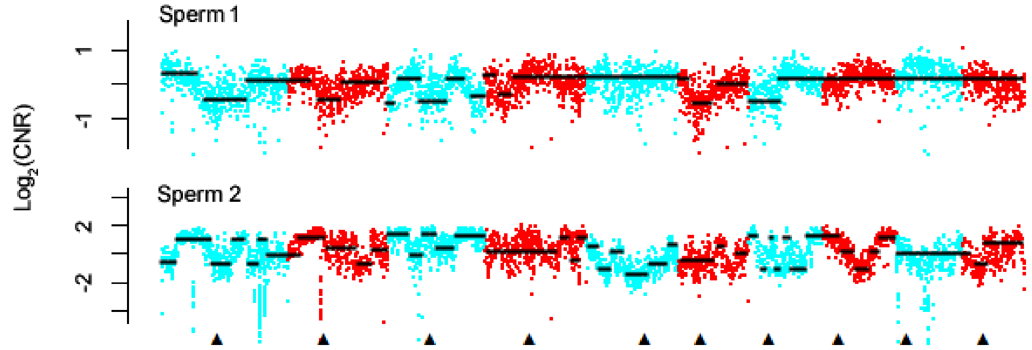

CHOI3 Pollen 35

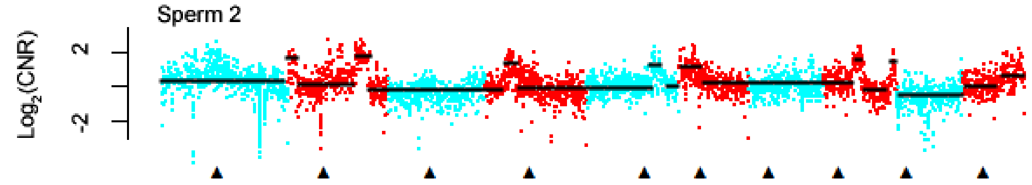

Supplementary Figure 6

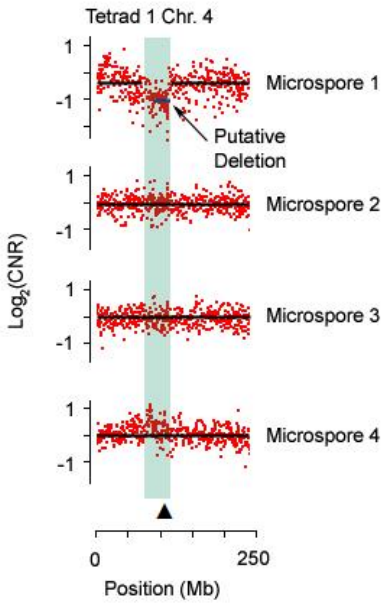

Supplementary Figure 6. A putative deletion exists in tetrad 1. The putative deletion region is shown in cold color background. The black and blue lines around spots indicate normal haploidy and deletion, respectively.

Supplementary Figure 7

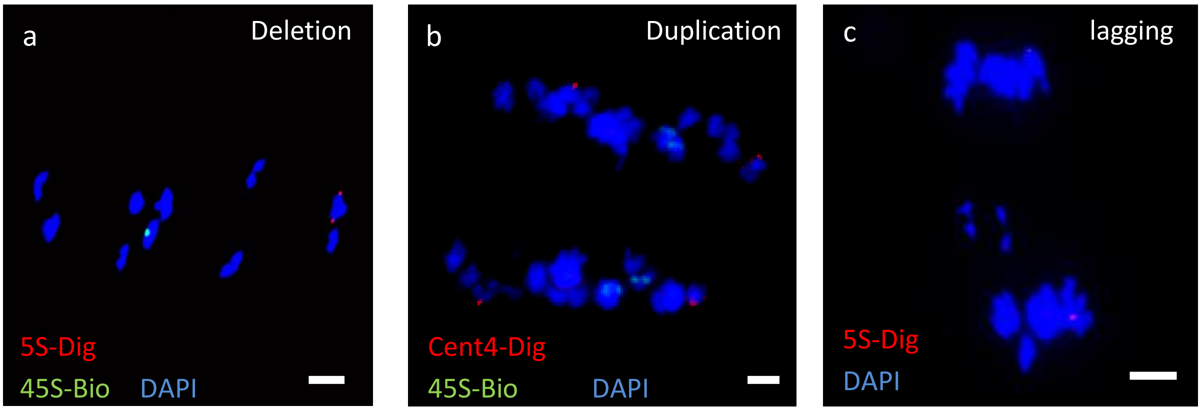

Supplementary Figure 7. Chromosome abnormalities of inducers. Deletion and Duplication chromosomes were probed with Cent4 (red), 5S (red), 45S (green). (**a**) Metaphase I chromosome of inducer CAU5 with one 45S signal (Deletion). (**b**) Duplication chromosomes of inducer CAU5 in anaphase I with four Cent4 and four 45S signals. (**c**) Four lagging chromosomes of inducer CAU5. Bars=10μm.
