# Supplementary material for: Single nucleus sequencing reveals spermatid chromosome fragmentation as a possible cause of maize haploid induction
Source: Nat Commun. 2017 Oct 23;8:991. doi: 10.1038/s41467-017-00969-8 (PMC5653662; doi:10.1038/s41467-017-00969-8)
Supplement: Supplementary file 3 — Description of Additional Supplementary Files [file 41467_2017_969_MOESM3_ESM.pdf]

Description of Additional Supplementary Files

File Name: Supplementary Data 1
Description: Rate of Chromosome lagging, deletion, duplication in meiotic process.

File Name: Supplementary Data 2
Description: Mapped reads numbers for CAU5 every single nucleus and cell analysed.

File Name: Supplementary Data 3
Description: Mapped reads numbers for B73 single sperm nucleus analysed.

File Name: Supplementary Data 4
Description: Mapped reads numbers for B73-inducer single sperm nucleus analysed.

File Name: Supplementary Data 5
Description: Mapped reads numbers for Chang7-2 single sperm nucleus analysed.

File Name: Supplementary Data 6
Description: Mapped reads numbers for CHOI3 single sperm nucleus analysed.

File Name: Supplementary Data 7
Description: Windows defined for CNV calling of CAU5 single nucleus and cell sequencing data.

File Name: Supplementary Data 8
Description: Windows defined for CNV calling of B73 single nucleus sequencing data.

File Name: Supplementary Data 9
Description: Windows defined for CNV calling of B73-inducer single nucleus sequencing data.

File Name: Supplementary Data 10
Description: Windows defined for CNV calling of Chang7-2 single nucleus sequencing data.

File Name: Supplementary Data 11
Description: Windows defined for CNV calling of CHOI3 single nucleus sequencing data.

File Name: Supplementary Data 12
Description: Mapped reads amount of every embryo and endosperm sequencing data.

File Name: Supplementary Data 13
Description: Windows defined for embryo and endosperm CNV calling.

File Name: Supplementary Data 14
Description: 700-SNPs contained windows defined for embryo and endosperm SNV ratio calling.
